# Obligate development of *Blastocrithidia papi* (Trypanosomatidae) in the Malpighian tubules of *Pyrrhocoris apterus* (Hemiptera) and coordination of host-parasite life cycles

**DOI:** 10.1371/journal.pone.0204467

**Published:** 2018-09-27

**Authors:** Alexander O. Frolov, Marina N. Malysheva, Anna I. Ganyukova, Vyacheslav Yurchenko, Alexei Y. Kostygov

**Affiliations:** 1 Zoological Institute of the Russian Academy of Sciences, St. Petersburg, Russia; 2 Life Science Research Centre, Faculty of Science, University of Ostrava, Ostrava, Czechia; 3 Biology Centre, Institute of Parasitology, Czech Academy of Sciences, České Budejovice (Budweis), Czechia; 4 Martsinovsky Institute of Medical Parasitology, Tropical and Vector Borne Diseases, Sechenov University, Moscow, Russia; University of Veterinary Medicine Vienna, AUSTRIA

## Abstract

*Blastocrithidia papi* is a unique trypanosomatid in that its life cycle is synchronized with that of its host, and includes an obligate stage of development in Malpighian tubules (MTs). This occurs in firebugs, which exited the winter diapause. In the short period, preceding the mating of overwintered insects, the flagellates penetrate MTs of the host, multiply attached to the epithelial surface with their flagella, and start forming cyst-like amastigotes (CLAs) in large agglomerates. By the moment of oviposition, a large number of CLAs are already available in the rectum. They are discharged on the eggs' surface with feces, used for transmission of bugs' symbiotic bacteria, which are compulsorily engulfed by the newly hatched nymphs along with the CLAs. The obligate development of *B*. *papi* in MTs is definitely linked to the life cycle synchronization. The absence of peristalsis allow the trypanosomatids to accumulate and form dense CLA-forming subpopulations, whereas the lack of peritrophic structures facilitates the extensive discharge of CLAs directly into the hindgut lumen. The massive release of CLAs associated with oviposition is indispensable for maximization of the infection efficiency at the most favorable time point.

## Introduction

The flagellates of the family Trypanosomatidae are worldwide-distributed obligate parasites of a wide range of invertebrates, vertebrates, and plants [1–3]. Depending on the number of hosts involved in their life cycle (one or two), trypanosomatids are divided into monoxenous and dixenous, respectively. In contrast to the dixenous relatives, which include agents of severe diseases of humans, domestic animals, and plants, the monoxenous trypanosomatids were overlooked for a long time and our knowledge about them is still somewhat limited [4, 5]. The molecular phylogenetic studies have demonstrated that the previous views on the diversity, taxonomy and evolution of this group were mainly incorrect. A number of traditional genera turned out to be polyphyletic [6–8]. Many new genera were erected to reflect the complex phylogenetic relationships within Trypanosomatidae and growing appreciation of their diversity. In addition, larger clades (considered in the current classification as subfamilies) were characterized [3, 9]. One of such groups, uniting the so-called cyst-forming trypanosomatids [10, 11], did not receive any taxonomical name yet. It encompasses the members of two formal genera still to be revised: *Blastocrithidia* and polyphyletic *Leptomonas* (only species related to *Leptomonas jaculum*). These flagellates are distinct from others in their ability to form a specialized resting stage–cyst-like amastigotes (CLAs), adapted to long-term survival in the environment [12–14]. All species of this group known to date were described from true bugs (order Hemiptera) [2, 11, 15, 16]. The CLAs can survive outside the host for over 10 years in laboratory conditions and are able to withstand both low and high temperatures (from that of liquid nitrogen to + 60°C), treatment with detergents and desiccation [17–20]. The CLAs function as cysts, but structurally are different from them [21]. Numerous ultrastructural studies demonstrated that these cells do not have any extracellular envelope, the inherent feature of protists’ cyst stages [22–28]. Instead, CLAs are protected by the thick layer of specialized submembranous cytoplasm [21].

The modes of CLAs' differentiation vary in different representatives of cyst-forming trypanosomatids [29]. The most common and well-studied mode is the formation of the so-called "flagellar cysts" or "strap-hangers". These names reflect the fact that the predecessors of CLAs are formed via unequal division and remain attached to the flagellum of their mother cell for a long time [30].

In general, the life cycles of these trypanosomatids are poorly characterized. However, there are some exceptions: *Blastocrithidia triatomae* parasitizing triatomine bugs, *Leptomonas wallacei* from the lygaeid bug *Oncopeltus fasciatus*, and *B*. *papi* from the firebug *Pyrrhocoris apterus* [12, 15, 31]. *Blastocrithidia triatomae* was extensively studied because of its pathogenic effect on the insect hosts, which transmit Chagas disease [31, 32]. This species develops in midgut, hindgut, and Malpighian tubules (MTs). The CLAs are formed in the rectum of the bug host and undergo horizontal transmission by coprophagy [33]. In MTs, some flagellates attach to the epithelial brush border without forming aggregates [34]. *Leptomonas wallacei* inhabits the host's midgut and hindgut, forming CLAs in the host rectum. The parasite is transmitted horizontally by coprophagy or vertically via eggs’ surface contamination [12]. The most recently described species, *B*. *papi* parasitizing intestine and MTs, uses CLAs for transmission by the same mechanisms [15].

Using the experiments with artificial diapause interruption, light and electron microscopy, we demonstrated that the development of *B*. *papi* in MTs is obligatory and coordinated with the life cycle of its host. We also described fine mechanisms of interaction between the flagellate and the host epithelium, as well as details of CLAs' formation process.

## Material and methods

### Hosts

The firebugs, *Pyrrhocoris apterus*, were collected in 2015–2017 from a naturally infected colony in the north of Pskov region, Russia (58°35'N; 28°55'E) [35]. No specific permissions were required for the insects' sampling, since the location of the study is of public access and *P*. *apterus* is not an endangered or protected species. The insects were either dissected immediately after their capture or maintained as laboratory culture.

### Experimental interruption of insects' diapause

The diapausing individuals of *P*. *apterus* were manually collected in mid-October from the leaf litter covered with snow below a linden tree in the center of the colony area. The firebugs were first maintained for one week at +10°C and 12/12 hours light cycle in a plastic container with leaf litter and a water bowl. After that, they were distributed over cages (20–30 individuals in each) with water bowls and food (ground linden seeds) and maintained at +24°C and 18/6 hours light cycle. During the experiment, one or two individuals per day were dissected. The average percentage of firebugs with infected and uninfected MTs was estimated every two weeks (n = 20).

### Dissection of insects

**Fig 1:**
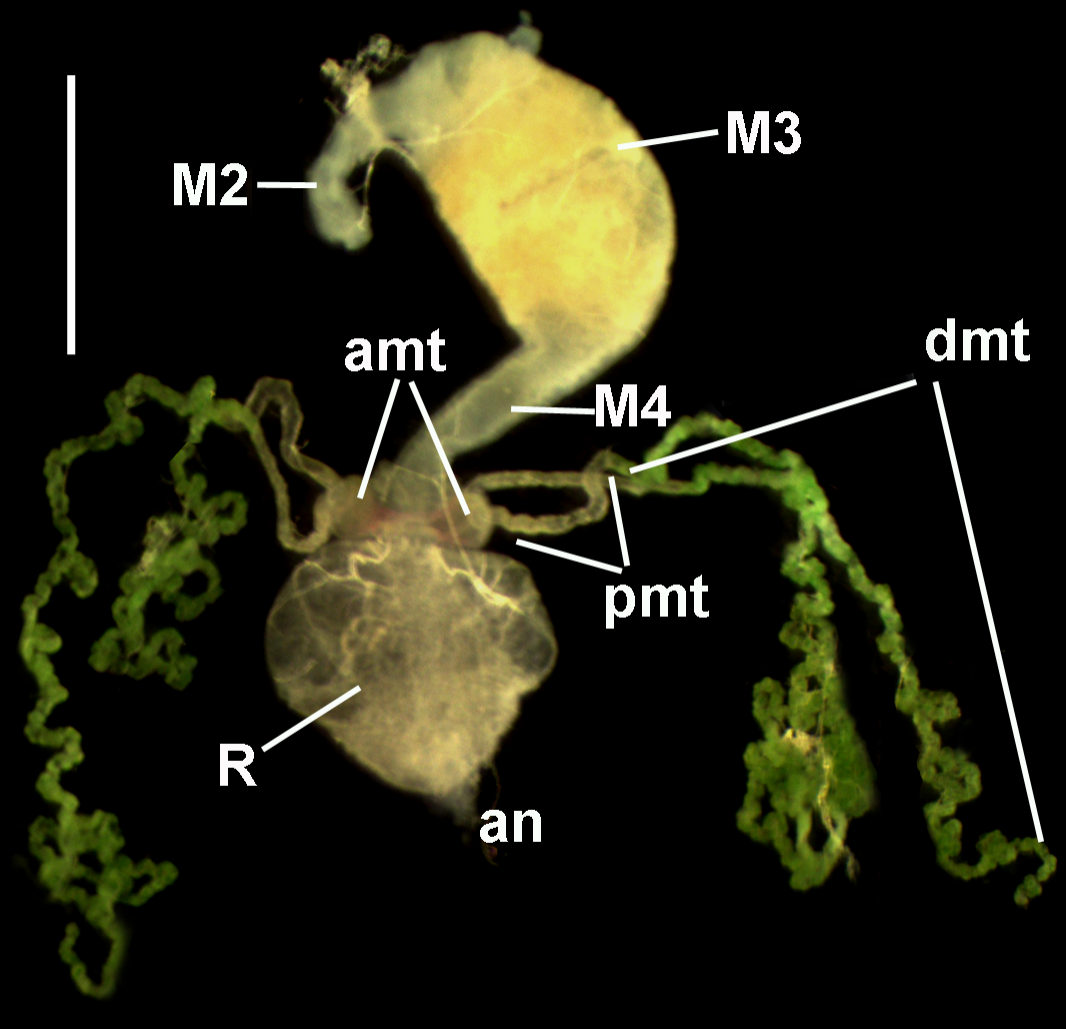
The posterior part of the digestive system of *P*. *apterus* with four MTs. **M2**, **M3**, and **M4** indicate segments of the midgut; **amt**–ampullae of MTs; **dmt**–distal part of MT; **pmt**–proximal part of MT; **an**–anus; **R**–rectum. Reflected light microscopy. Scale bar– 1 mm.

The firebugs were euthanized with chloroform and dissected in normal saline solution under LOMO MBS-2 stereomicroscope (Micromed, Russia) using reflected light and equipped with UCMOS09000KPB 9-Mpx camera (Toup Tek, Hangzhou, China). The posterior part of the intestine (from M3 segment to rectum) with MTs was isolated (Fig 1) and examined by sequential excision under DM 2500 light microscope (Leica Microsystems GmbH, Wetzlar, Germany) at 400× magnification.

### Light microscopy

The smears were prepared according to the previously published protocol [36] and stained with either Giemsa or 4',6-diamidino-2-phenylindole (DAPI) as described before [37, 38]. Digital images were acquired in DM 2500 microscope (Leica Microsystems GmbH, Wetzlar, Germany) equipped with UCMOS14000KPA 14-Mpx camera (Toup Tek, Hangzhou, China) at ×1,000 magnification. Giemsa-stained smears were observed and photographed in bright field (BF), living cells using differential interference contrast (DIC), whereas DAPI-stained smears with both DIC and fluorescence microscopy. All measurements of cells (n = 31) and statistical analysis were performed in UTHSCSA Image Tool for Windows v. 3.0.

### Transmission and scanning electron microscopy

The fragments of infected organs were fixed with a cold mixture of 1.5% glutaraldehyde and 1.5% OsO_4_ in 0.1 М cacodylate buffer (pH 7.2) for 1 hour. For transmission electron microscopy (TEM), the material was then post-fixed with 2% OsO_4_ in 0.1 М cacodylate buffer (pH 7.2) on melting ice, dehydrated in an ascending ethanol series followed by propylene oxide and finally embedded in Epon-Araldite resin (Sigma-Aldrich, St. Louis, MO, USA). Ultrathin sections (60 nm) were prepared using an EM UC6 ultramicrotome (Leica Microsystems). The sections were contrasted with water solutions of uranyl acetate (for 2 hours) and lead citrate (for 5 minutes) and examined under Morgagni 268-D microscope (FEI Company/Thermo Fisher Scientific, Hillsboro, OR, USA) using 80.00 kV accelerating voltage. For scanning electron microscopy (SEM), the fixed and dehydrated material was processed in a critical point dryer HCP- 2 (Hitachi Ltd., Tokyo, Japan) and after coating with 20-nm layer of platinum in IB-5 Ion coater (Tokyo Giko Co. Ltd., Tokyo, Japan) was examined under a Quanta 250 microscope (FEI Company/Thermo Fisher Scientific) with 25.00 kV voltage.

## Results

The developmental cycle of *P*. *apterus* in the studied colony included a long winter diapause lasting up to 7 months (from mid-October to May). The firebugs overwintered as imagines and appeared at the end of April. In the beginning of May, they mated and, in about two weeks, started laying eggs. The overwintered generation died by the end of June. Nymphs appeared in June, and after a series of molts, they transformed to imagines at the end of July–beginning of August. This new generation did not reproduce and was active until the mid-October, when bugs entered diapause, thus finishing the annual cycle.

### Dynamics of MTs' infection

The dissections of various instars of firebugs hatched in the current year were performed from June to October during three seasons (2014–2016). In these insects, no CLAs were detected either in the digestive tract or in the MTs. However, such cells were regularly observed in the MTs of imagines overwintered in the wild or maintained in laboratory. In the spring of 2016, we sampled the firebugs from the wild colony when they were i) exiting diapause (17 April) and ii) laying eggs (18–23 May). Comparison of their MTs' infection rates revealed that in the first case only 1 out of 30 individuals (3%) contained several epimastigotes of *B*. *papi*, whereas in the second case 41 out of 45 firebugs (91%) had infected MTs displaying massive formation of CLAs.

**Fig 2:**
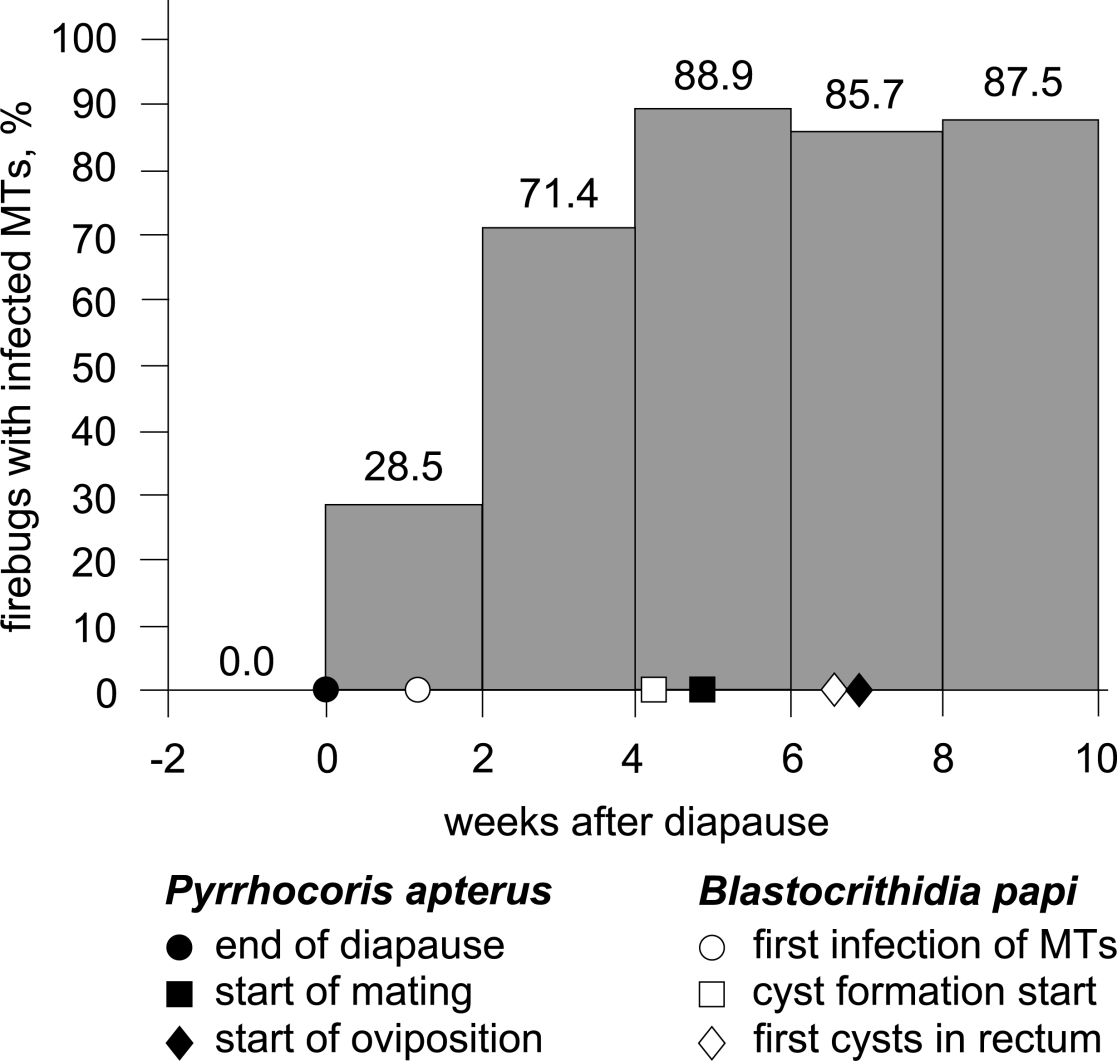
Dynamics of MTs' infection after experimental interruption of the winter diapause. Key events in the life cycles of the host and its parasite are indicated on the X-axis.

**Fig 3:**
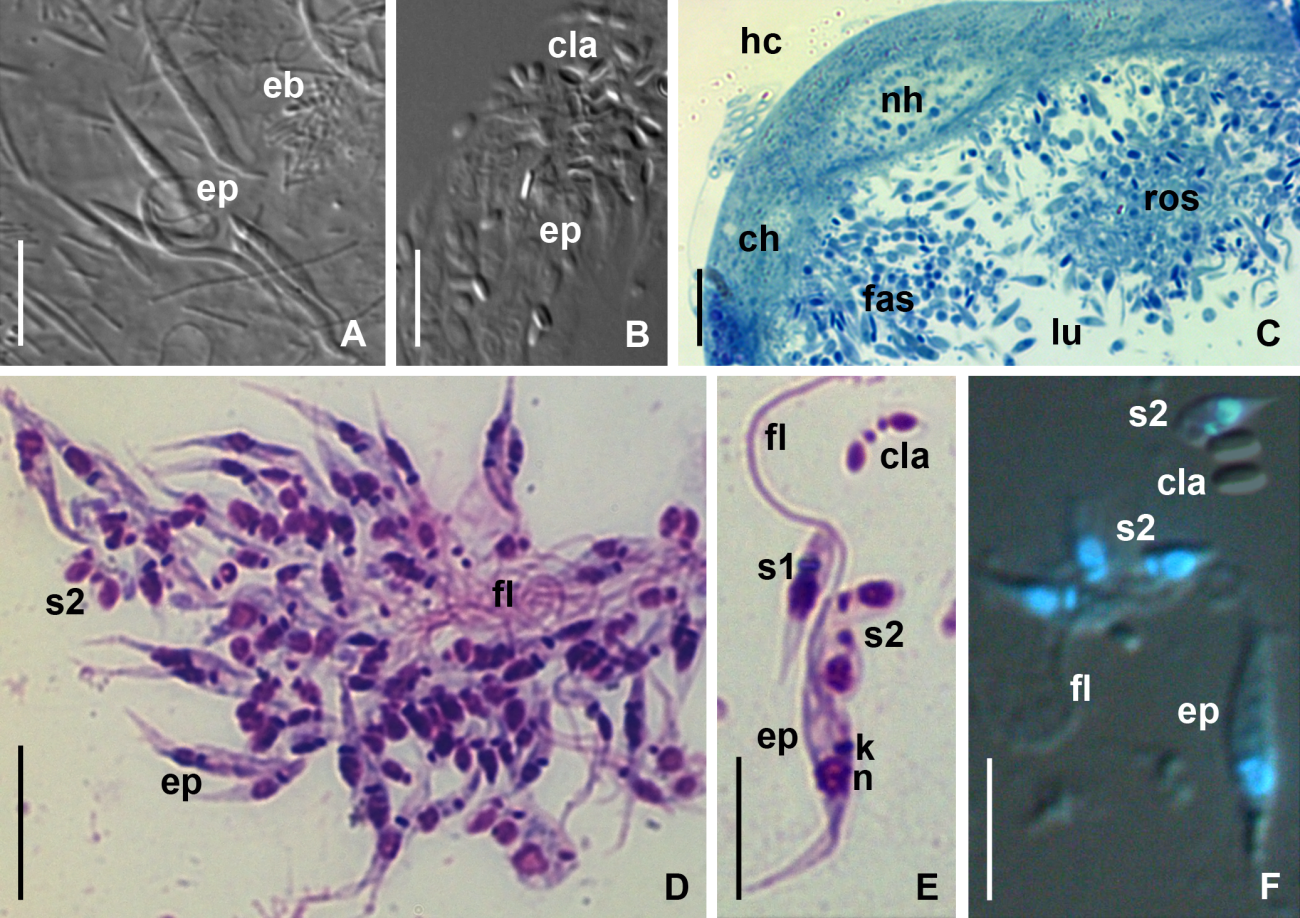
Light microscopy of *B*. *papi* in the intestine and MTs. (A) Epimastigotes in the M3 segment of midgut of diapausing *P*. *apterus* (DIC). (B) CLAs in the rectum of ovipositing female (DIC). (C) fragment of transverse semi-thin section of an infected MT (methylene blue staining, BF). (D) rosette-like aggregate (Giemsa-stained smear, BF). (E) Different stages of cyst formation (Giemsa-stained smear, BF). (F) combination of DIC and fluorescence microscopy illustrating the permeation of DAPI into all types of *B*. *papi* cells except mature CLAs. **ch**–cell of the host MT epithelium; **cla**–cyst-like amastigote; **eb**–prokaryotic endobionts; **ep**–epimastigote; **fas**–fascicule-like aggregate of flagellates; **fl**–flagellum; **hc**–hemocoel; **k**–kinetoplast; **lu**–lumen of MT; **n**–nucleus of parasite; **nh**–nucleus of host cell; **ros**–rosette-like aggregate of flagellates; **s1**, **s2** –stages of CLAs' formation. Other abbreviations are as in Fig 1. Scale bars: (A–C, E, F)– 10 μm; (D)– 15 μm.

In order to study the dynamics of МТ infection in more details, we set up an experiment with artificial interruption of diapause. Out of 15 examined diapausing firebugs, none had cells of *B*. *papi* in МТs (Fig 2), but all contained epimastigotes of this species in the M3 segment of the midgut along with prokaryotic microflora (Fig 3A). Most insects also contained promastigotes of *Leptomonas pyrrhocoris*, which exclusively inhabits lumen of the insect’s gut [39]. Given that *B*. *papi* was found attached to the intestinal epithelium and two co-infecting species were separated by the composite peritrophic matrix, we did not consider presence of *L*. *pyrrhocoris* relevant to the current investigation.

In the experiment, the first case of MTs infection was documented 8 days after the exit from diapause (a.e.d.). This specimen contained several epimastigotes in the distal part of one MT. During next two weeks, the number of firebugs with infected MTs increased progressively (Fig 2). However, the intensity of infection within this organ was low: rare epimastigotes in the lumen or small aggregates attached to the surface of epitheliocytes were detected. The flagellates were still numerous in M3, but were absent in M4 and rectum. Rare cells could be observed in the lumen of MTs' ampullae, paired sacs on the border between midgut and hindgut connected to proximal parts of MTs (Fig 1). In such cases, at least one associated MT was also infected.

Later, the proportion of insects with infected MTs considerably increased and reached the maximum of ~89% four weeks a.e.d. (Fig 2). This level was preserved until the end of the experiment. The intensity of the MTs infection gradually elevated resulting in their complete obstruction after 40 days of the experiment. The flagellates' penetration into the lumina of particular MTs and their initial localization within these organs appeared to be random. At 15–30 days a.e.d. (middle phase), the proximal (light), the distal (dark) and the both parts of MTs were infected with 30, 50 and 20% frequency, respectively. However, at that stage we never observed a generalized infection: affected regions alternated with intact ones without any visible regularity. At 40–60 days a.e.d. (late phase), 13, 40, 27, and 20% of the examined firebugs had one, two, three, and four MTs infected, respectively.

The formation of CLAs was first detected 30 days a.e.d. in large aggregates of epimastigotes, either attached to the epithelium or located freely in the lumen. The CLAs appeared in rectal content 45 days a.e.d. In three females bearing mature eggs, the rectum was entirely filled with "cysts", whereas all epimastigotes there were dead (Fig 3B).

The development of *B*. *papi* in MTs (including cyst formation) was studied with light and electron microscopy in the last experimental group of firebugs (45–60 days a.e.d.).

### Development of *B*. *papi* in MTs (light microscopy)

The morphometric comparison of epimastigotes from MTs and intestine revealed that the former are significantly shorter and possess longer flagella (Table 1). In MTs, the epimastigotes of *B*. *papi* formed large aggregates (Fig 3C and 3D), either free (rosette-like), or attached to the epithelium (fascicule-like). In the latter case, some cells were anchored to the MT wall, whereas the others interwove with them using long flagella (Fig 3C). In the aggregates of the both types, the cells were very active, often causing a perceptible pulsation of the infected MT wall. We suggest that rosette-like aggregates were secondary, formed after detachment of the fascicule-like ones.

**Table 1:** Morphometry of life cycle stages of *B*. *papi* in the MTs and midgut of *P*. *apterus*.

	Epimastigotes in the MT	Epimastigotes in the midgut	Strap-hangers S1 in MT	Strap-hangers S2 in MT	CLAs in MT
**Cell length**	16.3 ± 3.5 (10.6–25.4)	20.6 ± 5.0 (11.3–31.6)	8.5 ± 2.0 (5.7–14.2)	5.2 ± 0.7 (4.0–6.5)	2.9 ± 0.4 (2.1–3.4)
**Cell width**	1.8 ± 0.3 (1.2–2.7)	1.8 ± 0.3 (1.3–2.4)	1.7 ± 0.3 (1.4–2.1)	1.6 ± 0.2 (1.4–2.0)	1.4 ± 0.1 (1.3–1.5)
**Nucleus length**	2.1 ± 0.4 (1.4–3.1)	2.0 ± 0.4 (1.2–3.0)	–	–	–
**Nucleus width**	1.1 ± 0.3 (0.5–2.2)	1.0 ± 0.2 (0.5–1.5)	–	–	–
**Anterior end to kinetoplast distance**	6.4 ± 2.1 (2.4–12.8)	6.5 ± 2.7 (2.1–14.1)	–	–	–
**Anterior end to nucleus distance**	7.9 ±1.9 (3.9–13.4)	8.4 ± 2.8 (3.4–14.4)	–	–	–
**Free flagellum length**	19.7 ± 4.8 (11.8–28.8)	14.7 ± 3.4 (9.0–24.0)	–	–	–

All measurements are in μm.

In both types of aggregates, the parasites developed similarly and formed CLAs (Fig 3D–3F). This process started from unequal division producing a smaller cell (S1) attached to the flagellum of mother epimastigote. Fission of S1 resulted in the formation of two non-dividing S2 cells (Fig 3E and 3F), which eventually lost the connection to the flagellum of the mother epimastigote and differentiated into CLAs (Fig 3B–3F). Discrimination of mature and immature "cysts" was challenging on Giemsa-stained slides, but turned out to be straightforward with DAPI, which could not permeate CLAs (Fig 3F).

### Host-parasite relationships (TEM and SEM)

**Fig 4:**
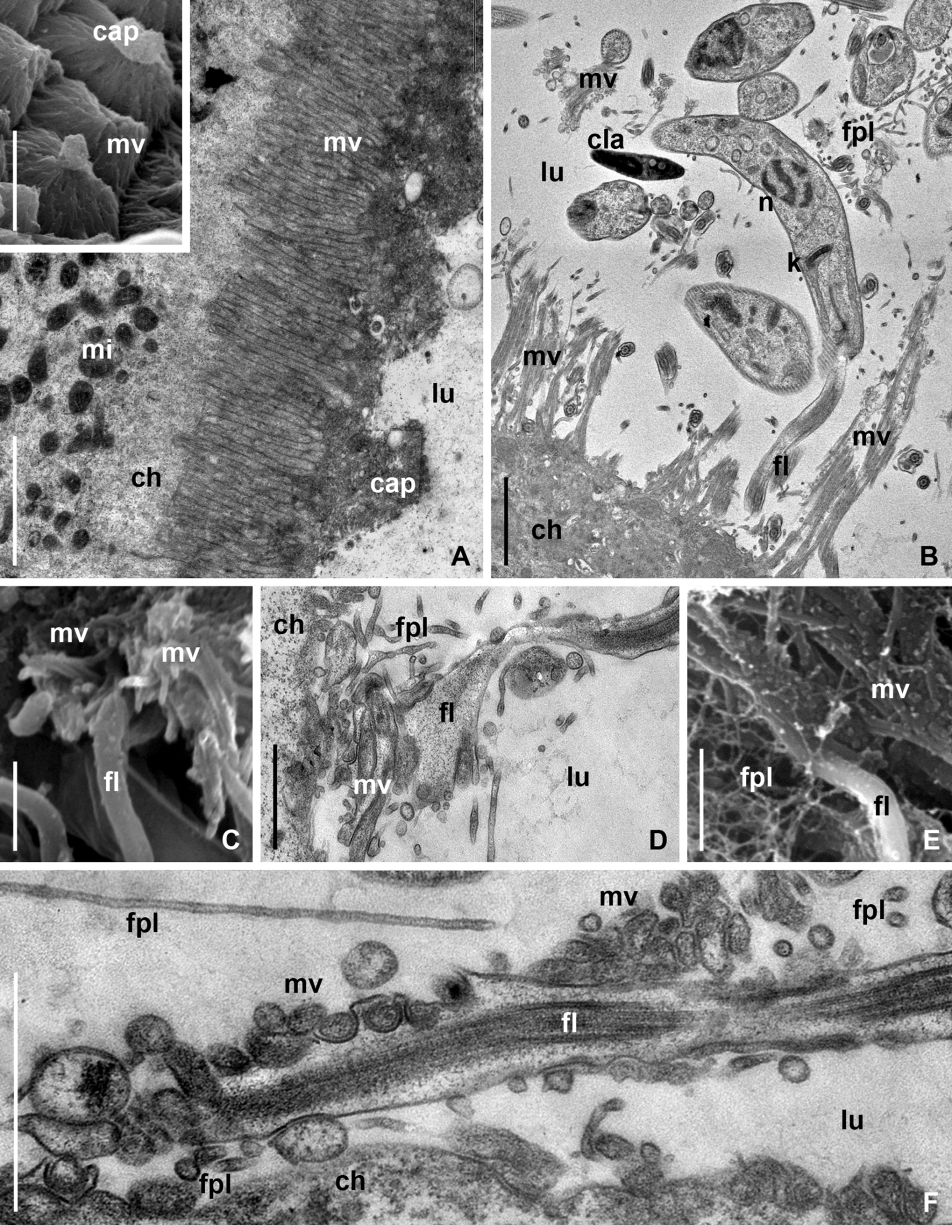
Development of *B*. *papi* in the MTs of *P*. *apterus* (TEM and SEM). (A) Brush border structure of intact МТ (TEM), inset–SEM. (B) Degradation of brush border of infected МТ in the same firebug as in A (TEM). (С –F) Attachment of parasites to brush border ((C, E)–SEM; (D, F)–TEM). **cap**–conglomerate of excretory products on the apical surface of microvillar papillae; **fpl**–filopodium-like appendages; **mi**–host cell mitochondrion; **mv**–microvilli. Other abbreviations are as in Figs 1 and 3. Scale bar: (А, B)– 2 μm; inset– 2,5 μm; (C–F)– 1 μm.

The intact epithelium of MTs in *P*. *apterus* consisted of large cuboidal cells bearing on the apical surface a brush border formed by closely spaced microvilli (Fig 4A). The latter were organized into regular conical assemblages (papillae) covered with characteristic "caps", representing conglomerates of excretory products, such as membrane-bound vesicles, concretions, etc. (Fig 4A, inset). In the infected regions of MTs, the brush border was completely absent or represented by scarce groups of microvilli (Fig 4B).

**Fig 5:**
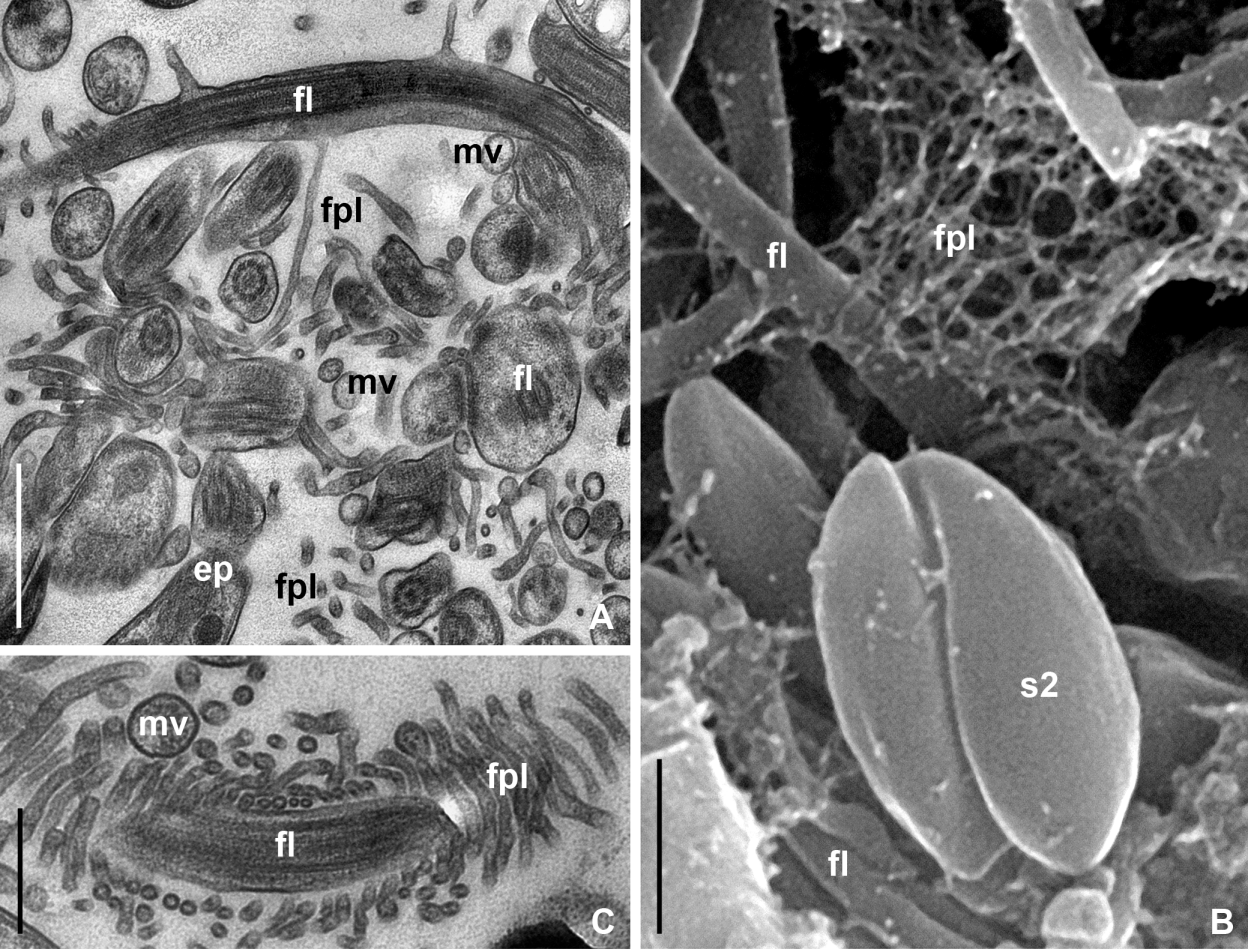
Organization of rosette-like epimastigote aggregates in the lumen of МТ (TEM and SEM). (A, B) Intertwining flagella and filopodium-like appendages in the center of an aggregate. (С) Epimastigote flagellum and microvillus both entwisted by filopodium-like appendages in the center of a rosette-like aggregate. All abbreviations are as in Figs 1, 3 and 4. Scale bars: (A, C)– 1 μm; (B)– 0,5 μm.

The process of the brush border degradation could be easily unraveled. At the early stage, the parasites destroyed the papillae by intruding there flagellar tips (Fig 4C), which could enlarge and seize individual microvilli or their groups (Fig 4D). In addition, the flagella penetrating into the brush border produced numerous thin (40–70 nm in diameter) tubular filopodia-like appendages entwisting host's microvilli and flagella of other epimastigotes (Fig 4E and 4F). The same appendages were observed in the central part of the rosette-like aggregates, where they additionally interconnected the entangled flagella (Fig 5).

The detachment of fascicule-like aggregates led to appearance of single or grouped microvilli in the lumen of infected MTs and in the central part of the flagellates' rosettes (Fig 5A). In contrast, uninfected MTs preserved all microvillar papillae and no fragments of brush border were detected in the lumen.

### Ultrastructure of CLAs formation in *B*. *papi*

**Fig 6:**
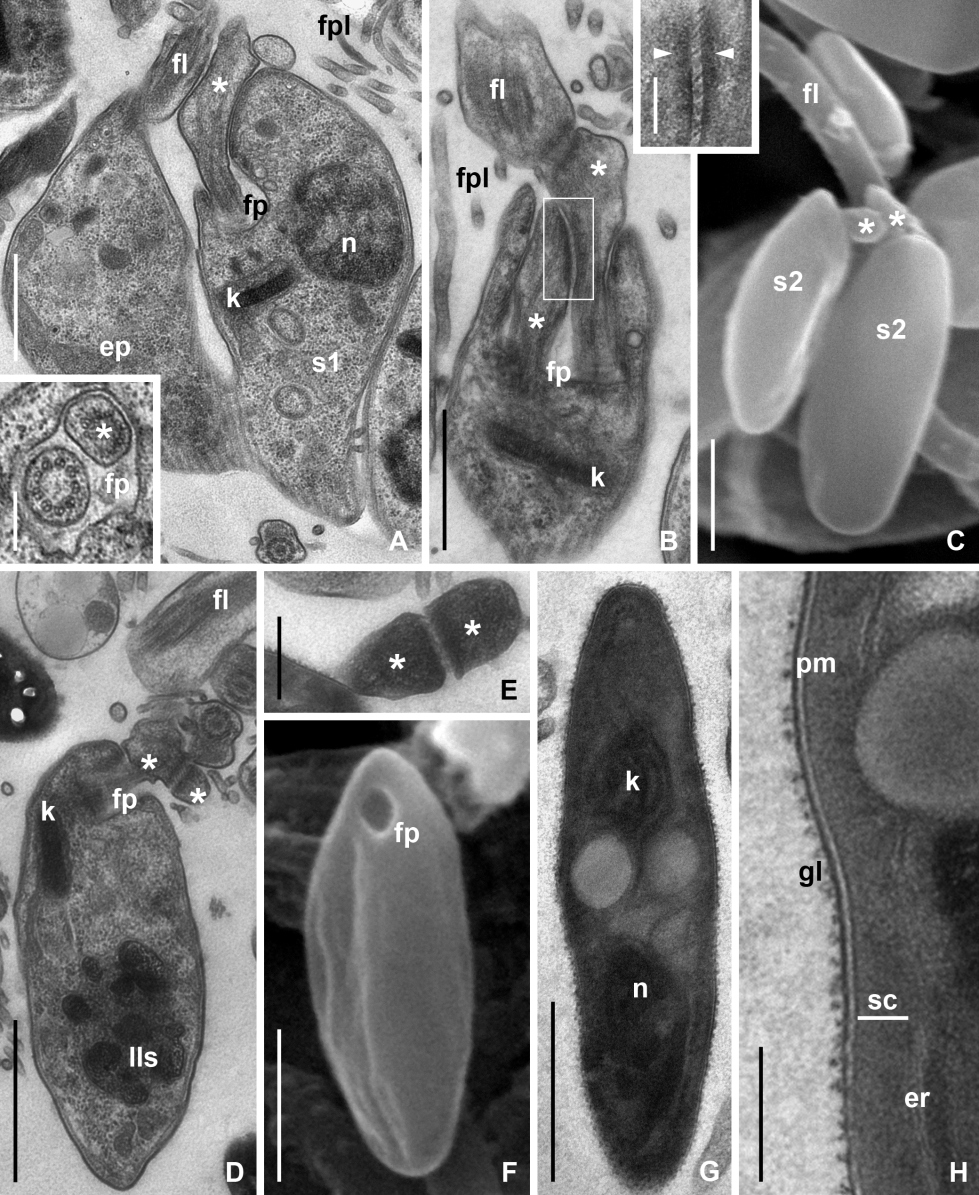
Formation of CLAs (TEM and SEM). (A) Unequal division of mother epimastigote (TEM), reduced flagellum of daughter cell (S1) without typical axoneme (inset). (В) Beginning of S1 cell division (TEM) showing attachment of the newly formed flagellum and the existing one using zonal desmosomes and fine transmembrane filaments (inset). (C, D) S2 cells preserving flagellar connection to each other and to mother epimastigote (SEM and TEM, respectively). (E) Flagella of S2 cells discarded during formation of CLAs and still attached to each other (TEM). (F) Young CLA with empty flagellar pocket (SEM). (G) General view of mature CLA (TEM). (H) Peripheral part of mature CLA (TEM). **er**–endoplasmic reticulum; **fp**–flagellar pocket; **gl**–glycocalyx; **lls**–labyrinth-like structure formed by chromatin; **pm**–plasmatic membrane; **sc**–layer of dense fine granular submembrane cytoplasm. Other abbreviations are as in Figs 1, 3 and 4. Asterisks–rudimentary flagella of S1 or S2 cells. Arrowheads–zonal desmosomes. Scale bars: (A)– 1 μm, inset 0.2 μm; (B)– 1 μm, inset 0.25 μm; (C)– 1 μm; (D)– 1 μm; (E)– 0.3 μm; (F)– 1 μm; (G)– 1 μm; (H)– 0.1 μm.

During the division of mother epimastigote most organelles were duplicated. However, the rudimentary flagellum of S1 cell contained neither paraxial rod nor typical axoneme (Fig 6A). The transverse section of the transition zone demonstrated disorganization of axonemal structure with replacement of some microtubule doublets by singlets (Fig 6A, inset). The flagellum of S1 was attached to that of the mother cell by the lateral surface of dilated tip (Fig 6A and 6B).

The subsequent division of S1 produced two S2 cells similar in size and flagellar structure (Fig 6B). One of them remained attached to the flagellum of the mother epimastigote, whereas its own flagellum was used to support another S2 cell (Fig 6B and 6C). Interestingly, during the division of S1, the new flagellum was growing connected to the already existing one (Fig 6B). While identification of mother and daughter S2 cells was not possible in light microscope (Fig 3E and 3F), electron microscopy allowed their unambiguous distinction by the order of flagellar connections (Fig 6C and 6D). In all cases, the attachment mechanism was the same: zonal desmosomes were formed at the site of contact and the slit-like space between the flagellar membranes contained fine transmembrane filaments (Fig 6B, inset).

Both S2 cells remained attached to the epimastigote for a certain time, during which their nuclei were undergoing considerable structural changes (Fig 6D). The condensed chromatin formed labyrinth-like structure (Fig 6D) typical of the nuclei in the majority of cyst-forming trypanosomatids [21]. Other changes associated with the maturation of CLAs happened after separation of S2 cells from the flagellum of the mother epimastigote (Fig 6F–6H). In contrast to the previously studied trypanosomatids, the last phase of CLAs' development, i.e. gradual condensation of organelles and formation of dense cortical layer of the cytoplasm [26] could not be observed, apparently due to the transience of this process. However, it was possible to document the stepwise separation of CLAs' predecessors. The pairs of S2 cells first detached from the mother epimastigote and then discarded their own flagella. The discarded flagella still preserved connection with each other and their content was rather dense in TEM (Fig 6E). Amastigotes with an empty flagellar pocket were found in proximity to these (Fig 6F). In mature CLAs, the flagellar pocket was absent (Fig 6G). These cells had characteristic organization (Fig 6G and 6H). Their surface was covered by loose glycocalyx. The plasmatic membrane had conspicuous electron-dense external outline. Under the membrane, there was a thick (up to 50 nm) layer of dense fine granular matrix with no subpellicular microtubules. The central part of the cell had dark zones corresponding to condensed nucleus and kinetoplast, spherical inclusions of medium electron density and small regions filled with tightly packed ribosomes (Fig 6G and 6H).

## Discussion

### The development of *B*. *papi* in Malpighian tubules is obligate

Most monoxenous trypanosomatids exclusively inhabit the insect intestine [5, 29]. Certain species can infect hemolymph and glandular organs associated with the digestive tract, such as salivary glands and Malpighian tubules [19, 25, 32, 35, 36, 40–43]. In some cases, the flagellates appear to be well adapted to this as judged by their ability to attach to host epitheliocytes and multiply. However, the biological implications of the development in extra-intestinal locations are not well understood, given that all key phases of the life cycle (including the formation of the resting stages) successfully take place in the intestine. The only exception to this is *Phytomonas nordicus*, whose development in hemolymph and salivary glands, indispensable for parasite transmission, stemmed from its dixenous ancestry [36].

The case of *B*. *papi* is unique. This is the only species known to date which is unable to complete its life cycle without infecting MTs. This organ is specifically used by this species for CLAs' formation. The absence of peristalsis allow the trypanosomatids to accumulate and form dense CLA-forming subpopulations, whereas the lack of peritrophic structures facilitates the extensive discharge of CLAs directly into the hindgut lumen. The importance of massive release of CLAs is elucidated below.

### Fine mechanisms of host-parasite interactions in MTs

Attachment was studied in detail for trypanosomatids inhabiting the insects' gut [29]. The most common mechanism used there is the anchoring of modified flagellar tips between the microvilli of enterocytes' brush border. The same mechanism was observed for the scarce studied cases of attachment in MTs. *Blastocrithidia triatomae* is able to seize the microvilli of MTs with the dilated flagellar tip and to induce invagination of the plasmatic membrane of the epitheliocytes [34]. In *Сrithidia flexonema* parasitizing the water strider *Gerris odontogaster*, the flagella interdigitate microvilli of host MTs' epitheliocytes and the inner leaflet of the flagellar membrane is thickened [44]. The epimastigotes of *B*. *gerridis*, a parasite of *Gerris lacustris*, are anchored in the MTs' brush border by shortened and swollen flagella [29].

The mode of attachment described here for *B*. *papi* is unique in several respects. The process starts from the destruction of papillae (regular microvillar bundles) by the flagella of individual epimastigotes. It has been recently demonstrated that structural stabilization of brush border in MTs of *Drosophila* is ensured by Fasciclin2 –mediated inter-microvillar homophile adhesion [45]. If the same mechanism of microvilli coalescence is employed in firebugs, the destruction of papillae by *B*. *papi* may be achieved not only by mechanically, but also enzymatically.

After the penetration of the parasite's flagellum, papilla opened like a flower bud releasing individual microvilli, which were then seized by the epimastigote's flagellar tip. In a similar way, the epimastigotes of *B*. *papi* attached to the walls of the midgut and *B*. *triatomae* in MTs of *Triatoma infestans* [15, 34]. However, in MTs of *P*. *apterus*, the epimastigotes of *B*. *papi* additionally formed multiple long tubular appendages, which entwisted each other, flagella of other individuals and microvilli of brush border. These thin tubules were usually formed by plasmatic membrane of flagella and, less frequently, other parts of the flagellate cell. Morphologically they are reminiscent of the filopodia of the bloodstream forms of African trypanosomes [46–48]. In monoxenous trypanosomatids, filopodia-like appendages were previously described in cyst-forming *Leptomonas wallacei* from the intestine of the milkweed bug *Oncopeltus fasciatus* and in *B*. *gerridis* from MTs of the water strider *Gerris lacustris* [28, 29]. Such appendages in *B*. *papi* appear important for the formation of flagellates' aggregates and their fixation to the surface of a host's MT. Cell divisions within an aggregate and inclusion of free epimastigotes from the lumen leads to the increased burden to the corresponding brush border region. This is aggravated by the active chaotic movement of the flagellates leading to the aggregate's jolting. These two factors play a crucial role in the abruption of the fascicule-like aggregate from the host brush border, leading to emergence of free "rosettes" and mechanical damage of the brush border. Brush border reduction in MTs was also described in nymphs of *Triatoma infestans* infected with *B*. *triatomae*. However, this was observed only upon long-term (~ 10 months) infection and was accompanied by extreme swelling of infected parts of MTs. In addition, epitheliocytes displayed accumulation of white concretions, reduction of mitochondria and basal cell interdigitation [34]. Such severe pathology was not observed in our study, apparently because of the relatively short period of MTs' infection.

### CLAs' formation

The mode of CLAs' formation through the development of "flagellar cysts" used by *B*. *papi* is the most widespread one and is known for all *Blastocrithidia* spp. of terrestrial bugs and some cyst-forming *Leptomonas* spp. from lygaeids [18, 23–26, 28, 49]. This process was well studied on light microscopy level, yet ultrastructural studies scrutinized mostly the different stages of CLAs' maturation. However, the details of divisions leading to the formation of CLAs, as well as the structure of the intermediate cells (S1 and S2) remained unclear. In this work, we were unable to follow the maturation process, but, instead, successfully filled the gaps of previous studies. Our most important finding in this respect concerns the fate of flagella in the intermediate stages. We demonstrated that every new cell attaches by its rudimentary flagellum to that of the preceding cell. Interestingly, even though S2 cells appear as products of equal division, one of them still can be considered maternal for the second one based on the attachment order.

### Host-parasite life cycle coordination

The life cycle of *B*. *papi* is well correlated to that of its host, *P*. *apterus*. In our study, the colonization of MTs by the trypanosomatid encompassed the period from the end of firebugs' diapause until the start of mating. At that time, both percentage of bugs with infected MTs and number of flagellates in a MT were increasing and they reached maxima 35–40 days a.e.d. From this moment started the formation of CLAs, and in 7–10 days these appeared in the rectum, ready to be discharged. This roughly coincided with the start of oviposition by female firebugs (day 46 a.e.d.). Importantly, while the M3 of the midgut segment was heavily populated already in nymphs, penetration to MTs occurred only in imagines, which entered the reproduction phase. In natural conditions, this is preceded by a long period of winter diapause of *P*. *apterus* [15, 35].

Coordination of host-parasite life cycles is well-known in some parasitic protists. It was documented that opalines living in frogs [50, 51] ciliates parasitizing frogs and insects [52–55], as well as gregarines inhabiting polychetes [56] correlate their development with the reproduction of hosts. Some insect gregarines coordinate their sexual process with insect pupation [56, 57]. Parabasalids parasitizing the intestine of long-lived cockroaches *Cryptocercus* spp. switch to sexual reproduction before host molting [58].

There are three main prerequisites for this phenomenon to appear: i) host specificity, ii) a distinct infective stage in the parasite life cycle, and iii) a particular moment in the host life cycle making the transmission possible or the most efficient. In the majority of cases, the coordination of life cycles occurs in parasites of animals with metamorphosis, such as insects or amphibians [59].

In trypanosomatids, coordination of the host and parasite life cycles has not been documented yet. Dixenous *Leishmania* and *Trypanosoma* apparently do not need such an adaptation. Indeed, these parasites spend most of the time in vertebrates, which provide long-term stable environment. The infection of vectors is restricted to the period of their blood-sucking activity, which, in the most cases, coincides with the relatively short adult phase. Therefore, the development in vectors must be quick in order to generate infective stages as soon as possible, i.e. before the next blood meal, and, again, no coordination is needed. The situation could be different in plant-dwelling *Phytomonas* spp., but the life cycles of these flagellates are largely unknown [60]. Provided their enormous diversity and restriction to insect hosts [4], monoxenous trypanosomatids would be expected to exhibit the phenomenon of life cycle coordination. However, many of them have wide host specificity precluding fine-tuned adaptations [61]. Moreover, the majority of studied monoxenous trypanosomatids do not have specialized infective stages and are transmitted using "vegetative" cells preserved in fresh feces, water, insect bodies, etc. In addition, only for sparse representatives of this group the life cycles were characterized.

*Blastocrithidia papi* is a unique example, where all the above requirements are met. The parasite is specific and possess a specialized infective stage–CLA. The specific habit of newly hatched bugs is the final factor of life cycle coordination determining the best timing for transmission: oviposition and subsequent eclosion. This habit consists in obligate engulfing of feces from the eggs' surface in order to establish their intestinal microflora and is also exploited by the parasite for its transmission [15, 62]. Therefore, CLAs must be available exactly at the period of oviposition. *B*. *papi* evolved to start massive CLAs production before this moment in order to ensure maximal infestation of new firebugs' generation. The obligate development in MTs (see above) appears especially important in this respect.

We propose that females of *P*. *apterus* enable mainly vertical ("transovum") transmission, while males are exclusively responsible for horizontal transmission by contaminating the colony area with their feces. The period of CLAs spreading is rather limited: it takes less than a month from the start of oviposition until the natural death of firebugs. Nevertheless, this period coincides with that of emergence of new generation of bugs, ensuring maximal infestation.
