# Correction: Obligate development of *Blastocrithidia papi* (Trypanosomatidae) in the Malpighian tubules of *Pyrrhocoris apterus* (Hemiptera) and coordination of host-parasite life cycles

**DOI:** 10.1371/journal.pone.0208178

**Published:** 2018-11-21

**Authors:** Alexander O. Frolov, Marina N. Malysheva, Anna I. Ganyukova, Vyacheslav Yurchenko, Alexei Y. Kostygov

In the Funding section, the grant number from the Russian Science Foundation is listed incorrectly. The correct number is: 18-14-00134.
